# Do large language models have a theory of mind?

**DOI:** 10.1073/pnas.2507080122

**Published:** 2025-07-03

**Authors:** Damian K. F. Pang, Severin K. Y. Pang, Marianne D. Broeker, Alix Hibble

**Affiliations:** ^a^Department of Experimental Psychology, University of Oxford, Oxford OX2 6GG, United Kingdom; ^b^School of Computer Science, University of St. Andrews, St. Andrews KY16 9AJ, United Kingdom

Michal Kosinski’s recent study on theory of mind (ToM) tasks given to different large language models (LLMs) (1) is fascinating and offers many insights into the continued evolution and development of LLMs.

When testing ToM in animals, much ethological research has focused on differentiating “genuine” ToM from other cognitive functions. *Morgan’s Canon* recommends using “lower” rather than “higher” psychological faculties to explain animal behavior where possible (2). While this “canon” may lack justification, “association-blindness” is also problematic (3).

Researchers working in developmental psychology and animal behavior have developed increasingly sophisticated methods to rule out alternative explanations (4), gradually building cumulative cases based on converging evidence (5). We suggest that the same should be done with LLMs. Kosinski considered some alternative explanations and included control trials to exclude simple heuristics (1). We suggest that this should be expanded by examining other alternatives like associative learning, which can be achieved through simple electronic circuits (6) and can be explicitly trained.

Given the sudden jump in performance in newer models, it is likely that LLMs have either been explicitly trained or engineered to solve ToM tasks, which could explain some observed differences from humans (7). Explicit training would likely result in overinferring false beliefs when a similar pattern exists. For example, stating that the container is transparent [inspired by the “goggles experiment” in ethology (8)] should not result in false beliefs while retaining a similar structure to ToM tasks. We suggest that wrongly inferring a false belief in such a scenario would be indicative of explicit training.

LLMs use mathematical representations of word vectors in a multidimensional space that include word associations and positions. Each vector is interpreted through surrounding vectors to give a broader context. Such structured composition can mimic the logic of its training data, given that logical relationships often result in specific vector patterns. LLMs are trained on texts created by humans as well as using reinforcement learning from human feedback (9). Both the training data and the feedback come from humans who possess a ToM, making it at least possible for LLMs to pass ToM tasks simply through pattern recognition.

Testing this would require ToM tasks with radically different patterns (not just novel particulars) from the ones found in the existing literature included in the training data. Alternatively, a significant improvement in ToM task performance in older models through training without model tuning (10) would indicate that patterns in the training data rather than in the model can account for task performance.

None of this implies that LLMs cannot have a genuine ToM. However, we propose that successfully solving isolated ToM tasks is insufficient evidence to indicate the presence of ToM (5). While the studies conducted by Kosinski (1) and others (7) are important and relevant, we suggest that attributing ToM to LLMs may be premature until simpler explanations can be ruled out and a cumulative case based on converging evidence can be made.
